# Role of Point-of-Care Diagnostics in Lower- and Middle-Income Countries and Austere Environments

**DOI:** 10.3390/diagnostics13111941

**Published:** 2023-06-01

**Authors:** Feroz Abubaker Ganchi, Timothy Craig Hardcastle

**Affiliations:** 1 Department of Surgical Sciences, Nelson R Mandela School of Clinical Medicine, University of KwaZulu-Natal, Durban 4001, South Africa; ganchif@ukzn.ac.za; 2 Trauma and Burns Services, Inkosi Albert Luthuli Central Hospital, Mayville 4058, South Africa

**Keywords:** LMIC, diagnostic devices, surgical, medical, emergency, austere environment, ultrasound, laboratory, mobile

## Abstract

Introduction: Austere environments include the wilderness and many lower- and middle-income countries, with many of these countries facing unrest and war. The access to advanced diagnostic equipment is often unaffordable, even if available, and the equipment is often liable to break down. Methods: A short review paper examining the options available to medical professionals to undertake clinical and point-of-care diagnostic testing in resource-constrained environments that also illustrates the development of mobile advanced diagnostic equipment. The aim is to provide an overview of the spectrum and functionality of these devices beyond clinical acumen. Results: Details and examples of products covering all aspects of diagnostic testing are provided. Where relevant, reliability and cost implications are discussed. Conclusions: The review highlights the need for more cost-effective accessible and utilitarian products and devices that will bring cost-effective health care to many in lower- and middle-income or austere environments.

## 1. Introduction

Lower- and middle-income countries (LMICs) are almost synonymous with being resource-deprived. It is probably no coincidence that many of these areas experience armed conflicts. This combination spells disaster for health care in these areas, further impacting the huge disparity in health care from higher-income countries (HICs). Access to healthcare in poorer countries is less than in wealthier ones. Even within countries, poorer people have less access than wealthier people [1].

Accurate yet rapid disease diagnosis, emergency resuscitation, and further management requires access to point-of-care (POC) diagnostic services. When POC testing services are accessible, health care is improved, especially in areas where health care infrastructure is weak, such as in LMICs. This is also the case where access to medical care is a challenge, as may be the case in an austere environment [1].

Bearing in mind several factors that will influence the accessibility of point-of-care diagnostics in LMICs, Desmond Kuupiel and coworkers suggest a lean and agile supply chain management framework [2]. The factors to be considered are: test selection, test quantification, procurement and storage, quality assurance, distribution of tests, and inventory management.

Tivani Mashamba–Thompson alluded to the idea that an assessment of current POC diagnostics should be conducted, with respect to the cost and quality of service being provided, before acquiring new services [3]. This review will briefly examine the role of point-of-care diagnostics in the following categories: history taking and clinical examination, monitoring vital signs, and imaging and laboratory-type analysis focusing on the austere environment and what is relevant to LMICs.

A number of factors will be discussed that affect the uptake and utility of POC devices, from the basics to more advanced aspects. This includes staff education on the basics of clinical acumen and the use of assistive POC devices to enhance the diagnostic capacity, rather than replace good clinical judgement. Then, the various devices and the capacity thereof will be covered.

## 2. Applying the Basics: History Taking and Clinical Examination

It must be emphasized that staff education and history-taking are paramount. All medical students at the beginning of the clinical years are informed that they will embark on being clinicians. In addition, about 80% of the diagnoses are made once a patient’s comprehensive history is recorded and a thorough examination is completed.

Clinical examination and history-taking are learned skills with many available books to guide the learner [4,5]. Further, the correct differential diagnosis can be achieved in most cases by combining history, signs, and symptoms. Unfortunately, comprehensive history-taking and clinical examinations are often no longer the norm today. The inclination towards, and reliance on, special investigations, partially as a result of an increase in litigation, has reduced the value of clinical acumen, to the detriment of patient care [6,7]. Therefore, an emphasis should be placed on educating clinicians working in resource-limited environments to optimize history-taking and clinical examination skills. These LMICs usually have fewer litigation risk levels. Then, medical students will develop the skill to appropriately and rationally use side-room and electronic point-of-care devices wisely and cost-effectively.

Good clinical examination will allow for focused imaging within the limitations of the accessible equipment. Sometimes clinical aspects will allow for the avoidance of unnecessary imaging. Examples of this include the various cervical spine imaging rules (e.g., Canadian C-spine rule or NEXUS) that will ensure that appropriately selected patients receive imaging when needed [8].

The chest is easy to evaluate clinically, and imaging can be guided by clinical findings suggesting a diagnosis (See Table 1).

A clinical assessment of the conscious patient allows for the identification of peritonitis, leading to operative intervention. Complete neurological examination allows for an assessment of higher function by establishing a sensory level or motor deficit using dermatome or myotome assessments. This will assist in establishing where the pathology is, meaning special investigations can be streamlined.

Becoming familiar with signs and symptoms pathognomonic for certain conditions will also expedite diagnoses and allow prompt management without unnecessary special investigations. For example, a lucid interval with a skull fracture on palpation and associated with pupil dilatation on the same side is most likely an *extradural* hemorrhage on the side of the fracture. This can allow focused imaging to guide surgery.

## 3. Vital Sign Monitors

State-of-the art multi-function vital sign monitors are used in most first-world emergency departments, or intensive care units. However, these are costly and also require constant electricity supply and regular maintenance. They are also not always portable. The advance in technology has made battery-operated, portable, and affordable vital sign monitors available.

The target market is usually what determines the quality of these instruments; home-based care devices are meant for low-volume usage, and the complexity of usage is minimal. Glucometers, blood pressure-measuring devices, and temperature-measuring devices are examples. A common mistake is procuring these for usage in other areas, such as peripheral clinics. Although they may be cheaper, their life span is very short if overused, and their reliability and reproducibility of accurate results is questionable [9,10].

Another dimension that has recently emerged is consumer-wearable digital devices. These devices are already being used by many consumers despite the lack of confirmatory testing in clinical settings. Christina Hahnen and coworkers tested two devices and found that both devices measured heart rate relatively accurately, but other vital signs were not as accurate [11]. Caution should be exercised if these devices are being used, especially in life-threatening situations.

Although a single monitor is probably the easiest manner in which to monitor a patient’s vital signs, COVID home-based care taught the world how to adapt. Battery operated isolated pulse-oximeters have proven to be a useful tool. In addition to providing a saturation level, they are usually accompanied by the measurement of the pulse rate. Plus, a battery-operated blood pressure monitor complements the monitoring of vital signs. Unfortunately, this combination excludes ECG monitoring, which is often required. The advantage of such a combination is in the simplicity of its use, with the relatively small price tag compared to high-tech equipment.

It can be concluded that in LMICs, vital-sign monitoring can be adequately performed across a spectrum, with the limiting factor being the availability of resources.

## 4. Plain Radiography

Plain radiographs are cheap, easily accessible, and usually available in LMICs, as well as austere environments on occasion. Limiting their use based on clinical findings will reduce unnecessary usage and also prolong the life span of the equipment used to acquire them. Being able to interpret plain radiographs supplements history-taking and clinical findings in attaining a final diagnosis. To derive maximal benefit from plain X-rays, a “reporting” system is recommended to work through each film, thereby increasing the ability to detect pathology and minimizing missing pathologies.

**Figure 1:**
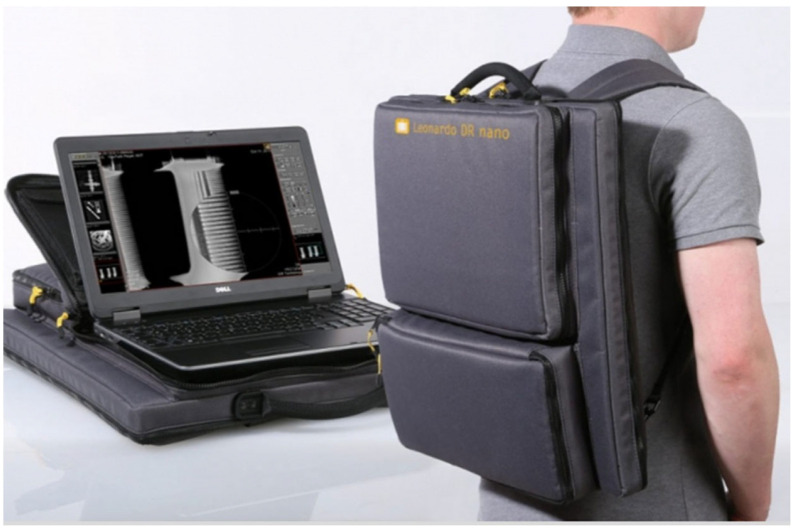
Image of a Leonardo nano mobile X-ray system.

Portable X-ray systems can now deliver good-quality images wirelessly to computers despite becoming smaller and cheaper. An example of this in Figure 1 is the Leonardo nano mobile X-ray system (https://www.or-technology.com/en/products/maritime/leonardo-dr-nano.html, accessed on 1 February 2023).

This entire X-ray system fits into a backpack. It is a portable, wireless X-ray system. It comprises only two components: a wireless detector, and a laptop with the professional acquisition and analysis software. Its image quality is superb. It can be used for both limb and torso images.

**Figure 2:**
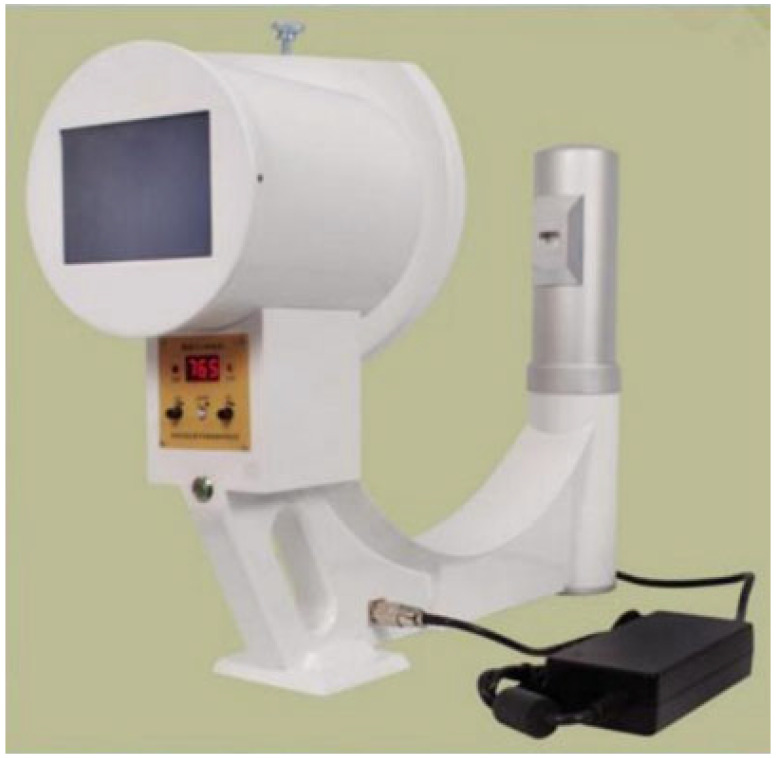
Mobile 1-piece X-ray system.

A system such as this would be ideal for taking services to communities in LMICs. However, its price tag is the limiting factor. Many military units are using this system, or an equivalent. Other devices, similar in concept yet less complex (and much cheaper), are also alternatives. See Figure 2 for an image of one such device.

An example of such a more cost-effective system is the Mobile 1-piece X-ray system (Novalion instrument company, Jiangsu, China). The advantages of a machine, as illustrated in Figure 2, are portability, low required amounts of electricity, and the ability to be powered by a battery–inverter combination. The image is displayed on the screen and negates the need for further processing. The disadvantages include lesser detailed image quality compared to standard devices, as well as less-than-ideal chest or abdominal X-ray acquisitions. However, this type of machine is definitely an option in LMICs and other austere environments where access to any imaging device is minimal.

**Figure 3:**
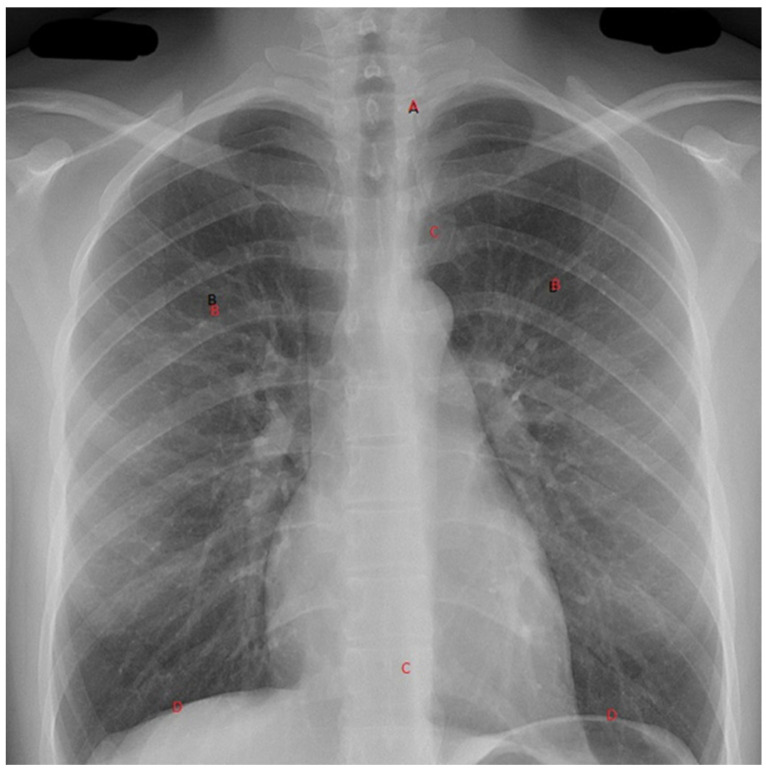
The ABDCEF approach to viewing a chest X-ray.

With these devices comes the need to ensure a reliable interpretation of the images obtained. Adopting a clinician-based reporting system to assess radiographs will allow an interpretation of these X-rays to optimize the identification of pathologies and the minimization of missed diagnoses. For **C-spine X-rays**, in interpreting a lateral c-spine X-ray, the following system may be adopted: adequacy (C1 to top of T1), check the alignment along four lines (namely: A, Anterior vertebral line; B, Anterior spinal line; C, Posterior spinal line; and D, Spinous processes). For the **Chest X-ray,** a recommended system for interpreting a plain AP Chest X-ray would follow an ABCDEF approach (illustrated in Figure 3): **A**irway, which is the position of the trachea and of an endotracheal tube if present; **B**reathing, which includes a lung-field assessment, looking specifically for haemothorax, pneumothorax, and contusions; **C**irculation, where mediastinal width, heart shape, and size are assessed; **D**iaphragms; any **E**xtras, including anything that is visible e.g., ECG electrodes, Nasogastric tubes, etc.; and **F**ractures, the examination of which follows a system from medially to laterally, from the ribs (anterior and posterior), to the clavicle, to the scapulae humerus, and to the vertebrae, if visible. A similar system can be used for the pelvis, limbs, and the vertebrae plain X-rays as well.

## 5. Ultrasound

Ultrasound usage has evolved from being a purely diagnostic instrument to assisting with interventions and reassessments post-intervention.

**Figure 4:**
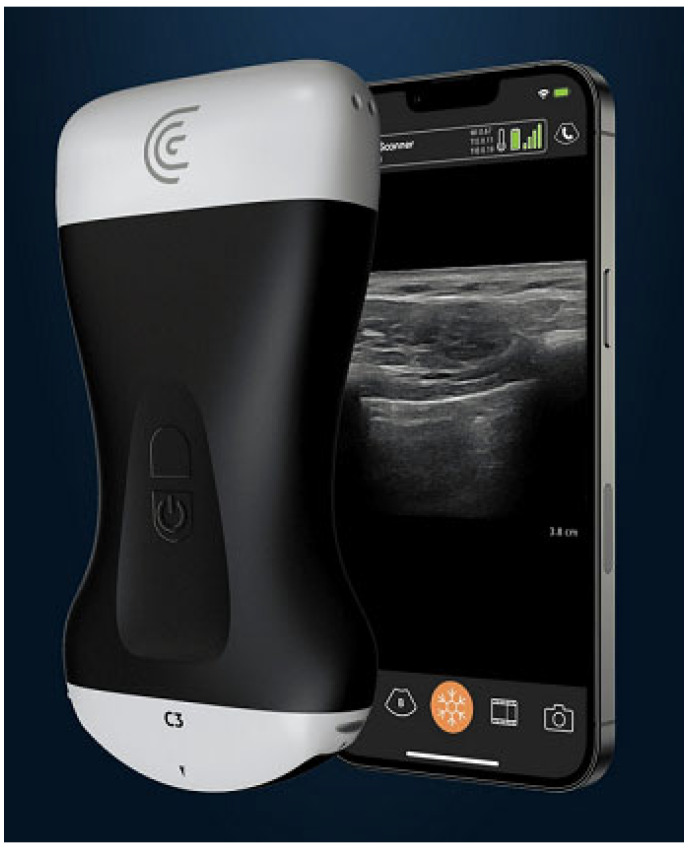
Image of ultrasound probe with linked mobile device onto which image is transmitted. There are a number of such devices, some with wire-to-the-phone, and others with Bluetooth^®^ or Wi-Fi connectivity.

Ultrasound machines have reduced in size but include better-quality resolution. Surprisingly, a mid-range handheld portable ultrasound probe, which can be linked to a mobile device, (telephone or tablet) is relatively inexpensive; one is illustrated in Figure 4 as an example. These portable probes are exceptionally useful in LMICs, and especially so in the austere environment. They can be transported and charged with ease.

Point-of-care ultrasound usage for diagnosis and guiding management in LMICs has become an option [12]. It does not necessarily require a specialized dedicated health care professional or dedicated infrastructure where it can be performed. In addition, it is a low-cost imaging modality [13].

Ultrasound devices have advanced to become more user-friendly. With appropriate knowledge and training, bed-side ultrasound examinations can be used to better diagnose pathologies and guide perioperative strategies. Cardiac ultrasound examination was the initial emphasis in anesthesiology, which has now expanded into lung and gastric ultrasound imaging [14].

### 5.1. Mass Casualty

Ultrasound usage in situations of mass casualties, especially in low-resource environments, can help with triage diagnosis re-evaluation if need be. This will assist in choosing an appropriate management plan in the prehospital setting. This modality of POC imaging should be integrated into protocols used by field-based practitioners [2].

### 5.2. Emergency Department

Point-of-care ultrasound (POCUS) is a useful tool in an emergency department. The role of POCUS is to rapidly assess and reach diagnoses in critically ill patients. Ultrasound probes that are small enough to be hand-carried and projected to mobile devices are useful in this setting; they offer the option for patient assessment, even in prehospital settings [15].

### 5.3. Trauma

The use of point-of-care ultrasounds in trauma has become commonplace. Focused Assessment with Sonography for Trauma examination is conducted bed side; this assists the clinician in deciding what the patient needs further and to which discipline they should be referred. This is a largely binary examination that aims to establish the presence, or lack thereof, of free fluid in the belly or pericardial sac. When performed by properly trained individuals, FAST is an accepted, rapid, and reliable study for identifying intraperitoneal fluid. It has the advantage of being repeatable and can also detect pericardial tamponade [16].

FAST includes the examination of four regions. The positive examination of unstable patients directs the operative approach, while in stable patients, it demands a further detailed assessment. The role of POCUS in trauma has expanded beyond the Focused Assessment with sonography for trauma examination. Advancements in diagnostics include contrast-enhanced ultrasounds, as well as the assessment of the chest and limbs. Ultrasound is also used in patients requiring vascular access or regional anesthesia. Its portability, affordability, and versatility have made ultrasounds an invaluable tool in trauma management in resource-limited settings [17].

### 5.4. Obstetrics

The obstetric anesthesiologist is able to assess the patient’s cardiac, pulmonary, neuraxial, gastrointestinal, and respiratory systems, and they can obtain a rapid diagnosis, as well as decide management in patients with common maternal peripartum comorbidities and obstetric complications. POCUS will aid midwives and obstetricians in the multidisciplinary practice on labour and delivery [18]. The role of ultrasounds in the diagnosis of pregnancy, the determination of gestational age, and fetal cardiac monitoring is well-established. They can provide important and potentially life-saving information. Routine ultrasounds provide information about the gestational age of the pregnancy and the fetal heart rate [19].

### 5.5. Intensive Care

Point-of-care ultrasounds in the intensive care unit are important diagnostic tools. As results of this imaging modality are instant, decisions are made instantly, and changes are implemented instantly based on the findings. As a result, management is not delayed, and further complications are potentially minimized. Bedside procedures, such as central line placement, lumbar punctures, or thoracocentesis, are carried out under ultrasound guidance. Ultrasound evaluations of cardiac activity, the pleural cavity, and abdominal cavity are conducted without the need to move the machine to a dedicated area [20]. One such example is the measurement of inferior cava collapsibility in the assessment of shock, giving the clinician an indication of the volume status of the patient [20].

### 5.6. Vascular

The use of point-of-care ultrasounds (POCUSs) in the evaluation of vascular emergencies, including abdominal aortic aneurysm and deep-vein thrombosis, is well-established. Direct visualization of the vasculature via B-mode, colour Doppler, and pulsed-wave Doppler has been reported to have assisted in the diagnosis of the following: (1) An acute, post-catheterization thrombus of the proximal radial artery; (2) A complete, traumatic radial artery transection; (3) A forearm hematoma with active arterial extravasation; (4) A traumatic arteriovenous fistula; (5) An acute thrombosis of an artery bypass graft; and (6) An infected pseudoaneurysm. POCUS usage in vascular patients allowed for the rapid identification of pathology within both arteries and veins, and improved patient management and prevented potentially harmful complications [21].

### 5.7. Orthopedics

The use of ultrasounds in assessing the musculoskeletal system has become more common. Advantages include a lack of exposure to radiation, the non-invasiveness of ultrasounds, their wide availability, their cost-effectiveness, real-time results, and the ability to use them anywhere (for example, in a general outpatient clinic). They have the advantage over traditional X-rays in that they detect soft tissue injury in addition to fractures [22].

Diagnoses in orthopedics often require further imaging beyond history-taking, clinical examination, and plain radiographs. Orthopedic patients often require more than traditional history-taking, examination, and plain X-rays to confirm diagnoses. POCUS could play a role in this scenario for ruling out occult fractures, as well as diagnosing joint effusions and tendon ruptures. By aiding a speedy diagnosis, unnecessary immobilization is reduced, as are inpatient stays, while allowing the introduction of early mobilization, and, thus, a reduced harm to patients [23].

POCUS can effectively be used as an alternative imaging method to traditional X-rays, as it has a high sensitivity in identifying fracture characteristics, thereby making it highly sensitive in diagnosing long bone fractures [24,25]. Supplementing physical examination with POCUS significantly improves diagnostic accuracy for dislocations, proximal humeral fractures, and reduction confirmation [26].

### 5.8. Military

As was discussed above regarding trauma and orthopedics, military clinicians are also able to conduct focused examinations and detect fractures with acceptable sensitivity and specificity. POCUS in the hands of trained military clinicians has the potential to improve diagnostic accuracy and ultimately improve care for the combatant and civilian casualty [27,28].

### 5.9. Ultrasound Training

It is imperative that training in ultrasound usage needs to be incorporated into medical degree curriculums. The Toronto Addis Ababa Academic Collaboration in Emergency Medicine (TAAAC–EM) recently established an introductory POCUS rotation within the Emergency Medicine residency program at Addis Ababa University [29]. Results were very promising.

A POCUS training course with seven sessions (each 2 h long) with skills stations covering ultrasound applications for trauma (Focused Assessment with Sonography for Trauma (FAST) examination), obstetrics, vascular, soft tissue, regional anesthesia, focused echocardiography, and ultrasound guidance for procedures was introduced into the General Surgery Training Program in Seattle, Washington. Results yielded in surgical residents improved self-efficacy and confidence levels across a broad range of skills [30]. In South Africa, a national POCUS curriculum has been established with three levels of certification [31].

In Zambia, a family medicine residency included a point-of-care ultrasound (POCUS) training program. Residents found it to be very helpful in their clinical decision-making. These data support the idea that POCUS education needs to be included in residency education systems, especially in LMICs, which will in turn promote the usage of POCUS in low-resource settings globally [32]. However, adequate proctoring is essential [33].

## 6. Laboratory Devices

The initial establishment of laboratory services requires a number of aspects to be addressed, including: skilled staff (e.g., haemotology, biochemistry, pathology, etc.), a suitable physical structure, an appropriate location within the structure, and appropriate equipment, along with a system of specimen collection and processing, including resulting. Post-initiation, these also need regular maintenance and a constant, reliable power supply [34].

LMICs and austere environments are sometimes lacking in all three of these aspects, and end-users are sometimes left with making do with whatever is available. This is where reliable POC devices can largely assist the clinician through clinician-performed testing.

Bed-side machines, such as bed-side haemoglobinometers, are very useful. Although not as accurate as formal laboratory testing, using these for trend measurements in critically ill patients remains a useful option. These devices usually use single-use consumables, which need to be procured and used with specific devices, so the health technology services must be intimately involved with the clinician in obtaining these consumables. Strip and stick tests for common endocrine or microbiological testing are also useful in these environments. Here, accuracy and reliability may again be limiting factors, depending on whether they have been tested against a formal laboratory standard [35,36,37].

The use of point-of-care testing (POCT) in different clinical applications is justified because it allows the physician to get results sooner and, thus, diagnose sooner and implement appropriate management in a shorter time. However, the negative aspects should also be considered, and attempts made at correcting these as processes advance. The negative aspects include analytical imprecision compared to conventional laboratory equipment, inter-manufacturer variability, the risk of inappropriate use, a low level of global regulation, and higher costs compared with conventional laboratory testing [38].

**Figure 5:**
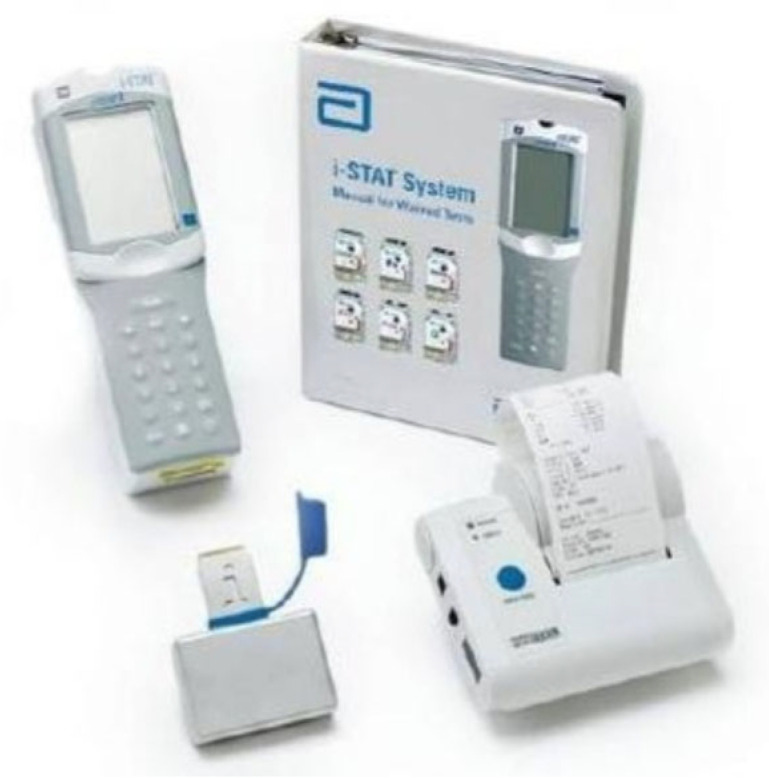
Image of complete iStat system (Abbott Laboratories, Chicago, IL, USA).

The military, and some civilian emergency medical services in higher-income countries, use state-of-the-art portable devices that can do almost any investigation, such as the iStat device often used by military medical teams operating in forward field projection points. This is illustrated in Figure 5 (https://www.globalpointofcare.abbott/en/index.html, accessed on 7 February 2023) (Figure 5). The machine itself is expensive, approximately USD 4500 for the system. The cartridges are what the limiting factor would be for LMICs, as these are expensive for repeated usage as an alternative to a fully fledged laboratory. However, where the fully fledged laboratory is unavailable, these POC devices may be lifesaving.

The iStat machine is able to do the following groups of investigations: blood gases, hematology, coagulation, chemistry and electrolytes, lactate, cardiac markers, and endocrinology. This analyzer is easy to use, reliable, and portable. Therefore, it is suitable for the operating room, for analyses during emergencies, on peripheral wards, for preclinical screening, or at times when the availability of lab tests is time-consuming or limited. The test accuracy for electrolytes, blood gases, and hemoglobin is high enough to justify a routine use of the i-STAT analyzer in clinical practice. While the nationally required quality standards for Calcium, pH, and hemoglobin were not met during testing, this is not of significance because the measured deviation was too small to have clinical relevance [39].

An alternative would be the Piccolo Xpress chemistry analyzer (https://www.globalpointofcare.abbott/en/index.html, accessed on 7 February 2023). This system offers the following: 31 blood-chemistry tests that range from liver, kidney, and metabolic functions to lipids, electrolytes, and other specialty analytes for routine testing, general health screening, and chronic conditions. These devices, and others from other suppliers, are robust and accurate in the results provided [40].

### 6.1. Microbiology

The rapid diagnosis of infectious diseases (in LMICs) within 2 h, 24 h a day, and 7 days a week is the driving factor in developing POC laboratories as an alternative to centrally based traditional laboratories. Their rapid turn-around time is largely based on immunochromatography, and real-time PCR tests that are conveniently combined into syndrome-based kits aimed at ease of use. The cost-effectiveness of POC laboratories has been established for the rapid diagnosis of tuberculosis and sexually transmitted infections in both HICs and LMICs [41].

### 6.2. Parasitology

Infectious diseases are more common in resource-limited environments and have the potential to have serious complications because they are transmissible. Therefore, early detection is paramount. Point-of-care testing provides an alternative to centralized laboratory testing. It has the advantage of being less time-consuming and while requiring fewer resources. Point-of-care test (POCT) usage has increased worldwide because it provides real-time rapid diagnosis. This facilitates the rapid initiation of treatment. Their use is especially beneficial in rural areas where parasitic infections are common and most needed. They are, unfortunately, not easily available, especially in low-resource settings where they are needed most. Despite the high demand, a relatively limited number of validated rapid diagnostics are commercially available for parasitic infections [42].

### 6.3. Intra-Operative

Perioperative POCT includes arterial blood gas monitoring, chemistry, CO_2_-oximetry panels, parathyroid hormone assays, and coagulation testing. Parathyroid hormone assays continue to guide surgical resections of the parathyroid glands. Point-of-care coagulation testing (such as viscoelastic assays) aids in the diagnosis of coagulopathy, as well as therapeutic optimization of anticoagulants such as clopidogrel and aspirin [43]. In modern resuscitation practices, cartridge-based clinicians performed thromboelastography (TEG™, (Haemoscope Corporation, Niles, IL, USA)) or rotational thrombelastometry (ROTEM^®^ (TEM Innovations GmbH, Munich, Germany)). This can be helpful in guiding resuscitation and blood-product transfusion. Several studies have demonstrated a reduction in the transfusion of blood components with TEG/ROTEM in both the civilian and military environments [44,45].

## 7. Challenges and Future Prospects

Technological advancements have changed the way diagnostics are conducted. More sensitive and more specific diagnostic modalities, with faster turnaround times (some with instant resulting) have emerged. Wealthy countries and military units have already implemented them. Unfortunately, they remain inaccessible to LMICs, which have less manufacturing capacity and less reliable quality assurance, thus creating a major challenge to advancement. A significant contributing factor in this regard is the supply demand dynamics and financial issues. With most up-to-date equipment currently being used by wealthier nations, those devices will be supplied to LMICs when newer items become available, superseding the older technology. Thus, LMICs are often at least one generation behind the HICs. This means they will eventually receive benefits from the newer developments, albeit after a delay. A suggestion to leaders of LMICs would be to try to narrow this gap. A starting point may well be addressing such issues in the BRICS countries of Brazil, Russia, India, China, South Africa, and possibly Saudi Arabia, where more capacity for local technology development and piloting exists, and where quality-of-care standards are largely adhered to.

## 8. Conclusions

There are multiple POC devices on the market that can increase access to diagnostics and enhance diagnosis times, both in austere environments, such as the wilderness or military sphere, and in LMICs with lower-income and technical challenges. Many devices are designed for use in these environments and cover the entire spectrum of care, from basic observations to complex diagnosis, including clinical assessment, imaging (radiology- and ultrasound-based), laboratory services, and operative care. Cost-effectiveness and availability, along with training, quality-assurance, and maintenance are the determining factors for uptake and resource implementation. The enhancement of manufacturing capacity in LMICs, and the development of healthcare technology and quality units in LMICs, will encourage use and improve clinical care.

## Figures and Tables

**Table 1:** Chest clinical features associated with trauma.

	Inspect	Palpate	Percuss	Auscultate
Pneumothorax	↓ chest movement	Trachea central	↑ resonance	↓ air entry
Tension pneumothorax	↓ chest movement	Trachea deviated away from side of pathology	↑ resonance	↓ air entry
Haemothorax	↓ chest movement	Trachea central	↓ resonance	↓ air entry
Massive haemothorax	↓ chest movement	Trachea deviated away from side of pathology	↓ resonance	↓ air entry

Up arrows—increased/down arrows—decreased clinical signs.

## Data Availability

The study did not report on any new data.
